# Reply to Jue et al. Value of MRI to Improve Deep Learning Model That Identifies High-Grade Prostate Cancer. Comment on “Gentile et al. Optimized Identification of High-Grade Prostate Cancer by Combining Different PSA Molecular Forms and PSA Density in a Deep Learning Model. *Diagnostics* 2021, *11*, 335”

**DOI:** 10.3390/diagnostics11071214

**Published:** 2021-07-06

**Authors:** Francesco Gentile, Matteo Ferro, Bartolomeo Della Ventura, Evelina La Civita, Antonietta Liotti, Michele Cennamo, Dario Bruzzese, Raffaele Velotta, Daniela Terracciano

**Affiliations:** 1 Department of Experimental and Clinical Medicine, University Magna Graecia of Catanzaro, 88100 Catanzaro, Italy; 2 Division of Urology, European Institute of Oncology (IEO), IRCCS, Via Ripamonti 435, 20141 Milan, Italy; matteo.ferro@ieo.it; 3 Department of Physics “Ettore Pancini”, University of Naples “Federico II”, Via Cintia 26 Ed. G, 80126 Naples, Italy; bartolomeo.dellaventura@unina.it (B.D.V.); rvelotta@unina.it (R.V.); 4 Department of Translational Medical Sciences, University of Naples “Federico II”, 80131 Naples, Italy; eva.lacivita@gmail.com (E.L.C.); antonietta.liotti@unina.it (A.L.); michelecennamo1@gmail.com (M.C.); 5 Department of Public Health, University of Naples “Federico II”, 80131 Naples, Italy; dario.bruzzese@unina.it

In their comment “Value of MRI to Improve Deep Learning Model That Identifies High-Grade Prostate Cancer. Comment on Gentile et al. Optimized Identification of High-Grade Prostate Cancer by Combining Different PSA Molecular Forms and PSA Density in a Deep Learning Model. Diagnostics 2021, 11, 335”, Jue and colleagues argued that, while artificial intelligence has the potential to revolutionize the detection of cancers and other pathologies in medicine, the use of PSA density in a deep learning model may be even more effective if, before the analysis, samples are stratified based on PSA values. Moreover, they stressed the importance of MRI in the diagnosis, treatment, and surveillance of localized prostate cancer. While Jue et al.’s PSA-based stratification suggestion is interesting, our model already intrinsically incorporates this effect. Sample stratification is an approach that can potentially improve the performance of the method. However, as it has been conceived, and for the very nature of machine learning, the algorithm automatically stratifies samples based on all of its inputs, including PSA. Notably, this ensures maximum performance for PSA ranges where the association between PSA values and clinically significant prostate cancer (csPCa) is clear and, in the same way, in PSA intervals where this association is uncertain, such as the 2–10 ng/mL range. Thus, a model that contains the variables used in our study, including PSA, is not entrusted with the skill of the operator; rather, it is quantitative, and less susceptible to errors. Moreover, the algorithm that we have developed is a general model where the number and type of input variables are not rigidly fixed. In this respect, our group struggled with the use of additional biomarkers for identification of clinically significant prostate cancer (PCa) and recognised a potential of PSA molecular forms that should not be dismissed [1]. We also noted that the model combining different biomarkers offers the potential for better diagnostic performance [2,3]. Thus, as clinical studies will confirm the relevance of other variables for PCa diagnosis, we will be able to include them in this model template, increasing even further sensitivity, specificity, and accuracy. Furthermore, a deep learning, multi parameter model could necessitate the inclusion of many patients for its correct implementation. The enrolment of many patients, which is ongoing, will enable us to test even further the model and its efficacy under a variety of different conditions and for different variables. There is general agreement that the main drawback of PSA’s widespread use is the overtreatment of organ-confined cancers. Overtreatment can be reduced by better algorithms based on the progressive use of tools able to identify high-risk PCa. Combined biomarkers have been demonstrated to be cost-effective [4] and might be one of the best ways to stratify patients at diagnosis. Other potential risk stratification strategies include mpMRI [5]. This technique offers an opportunity to focus radical prostatectomy only on those subjects who will truly benefit from it. However, there is evidence that the percentage of men with a positive mpMRI result who harbour csPCa was not 100%: it was observed in 83% of men with a PI-RADS score of 5, 60% of men with a score of 4, and only 12% of subjects with a score of 3, which corresponds to an uncertain outcome [6]. Thus, there is a non-negligible subset of patients in whom mpMRI yields overdiagnosis. As an alternative strategy, a panel of blood-based biomarkers does not carry the cost, inter-reader variability, and required expertise of mpMRI. Assessment of disease characteristics by a deep learning model from a blood sample is very likely to be useful to select the men who truly need mpMRI, as suggested by recently published studies [7]. Our work on a deep learning model based on PSA molecular forms moves this approach forward, and it has a great potential to be used to safely select men for active surveillance protocols. A challenge for the future will be how to deal with the increasing rate of PCa detection due to demographic changes. There will be a growing risk of overdiagnosis and overtreatment if the screening is based on t-PSA alone. Thus, it is mandatory to find ways to avoid unnecessary biopsy and proceed to surgery only for those men who are at risk of having aggressive PCa. Further studies in this area will probably refine blood-based biomarkers’ risk-adapted algorithms, and this will be essential in developing ways to minimize overtreatment without missing the identification of csPCa. The use of deep learning models to guide clinical management, as also recognized by Jue et al. “Value of MRI to Improve Deep Learning Model That Identifies High-Grade Prostate Cancer. Comment on Gentile et al. Optimized Identification of High-Grade Prostate Cancer by Combining Different PSA Molecular Forms and PSA Density in a Deep Learning Model. Diagnostics 2021, 11, 335” is essential for the development of a rational approach to significantly increase the number of men who can be safely managed by conservative methods. In such a context, deep learning models based on blood biomarkers may represent a cost-effective and widespread used tool to optimize the identification of high-grade PCa.
